# Editorial: Pathogen co-infections and plant diseases

**DOI:** 10.3389/fmicb.2023.1189476

**Published:** 2023-05-10

**Authors:** Jianuan Zhou, Steffen Kolb, Xiaofan Zhou

**Affiliations:** ^1^National Key Laboratory of Green Pesticide, Guangdong Province Key Laboratory of Microbial Signals and Disease Control, Integrative Microbiology Research Center, South China Agricultural University, Guangzhou, China; ^2^Microbial Biogeochemistry, Research Area Landscape Functioning, Leibniz Center for Agricultural Landscape Research e.V. (ZALF), Müncheberg, Germany

**Keywords:** plant diseases, co-infection, microbial community, interactions, synergism

Plant-microbe interaction has always been the focus of agricultural life science and has continuously increased the knowledge of human cognition about the nature. However, previous efforts in this regard have mostly focused on “one-to-one” binary relationships, such as the most classical plant-pathogen interaction “zigzag” model proposed by Jones and Dangl (2006). However, interactions between microbes are important components of the complexity of nature. With the development of microbe separation techniques and especially the metagenomic sequencing technology, communities of co-occurring pathogens are increasingly identified in diseased plants, and co-occurrence patterns and inter- and intra-species interactions have started to draw attention. Disease outbreaks may be incited by a certain mixture of microbes that are spatially aggregated in the field and migrate patch to patch in a short time (Burdon, 1993; Morris et al., 2017). Moreover, in reality, the distribution and composition of such microbe metapopulations are dynamic with changes in climate and environmental conditions.

In this special issue, four articles were published. Zhang et al. in their article “*Co-infection of Fusarium aglaonematis sp. nov. and Fusarium elaeidis causing stem rot in Aglaonema modestum in China*” obtained multiple *Fusarium* isolates from the diseased *A. modestum*, and determined *F. aglaonematis* and *F. elaeidis* belonging to different *Fusarium* species complex as the pathogens. Co-infection of the two species enhanced the disease severity of the host compared to single inoculation. Similar examples of synergistic interactions of pathogen-pathogen in plants that lead to increased disease severity have often been reported. For instance, co-infection of *F. oxysporum* f. sp. *medicaginis* and *Rhizoctonia solani* resulted in more severe disease incidence and growth reduction in commercial alfalfa production compared to a single infection (Fang et al., 2021). For tomato pith necrosis, co-infection of *Pseudomonas corrugate* with *P. marginalis* or *P. mediterranea* greatly enhanced the severity of the disease in tomato (Moura et al., 2005; Kudela et al., 2010). Co-inoculations with *Pythium* and *Fusarium* species caused increased disease symptoms compared to either pathogen alone (Lerch-Olson and Robertson, 2020). More commonly, different pathogens with imperceptible relationships have been purified from the same disease plant tissue, such as the recent studies identifying *Dickeya zeae* and *Stenotrophomonas maltophilia* as the soft rot pathogens on *Clivia minita* (Hu et al., 2021a), *Xanthomonas perforans* and *Pantoea ananatis* as the causal agents on water spinach (*Ipomoea aquatic*) (Hu et al., 2021b), and *Enterobacter asburiae* and *Pa. ananatis* causing rice bacterial blight in China (Xue et al., 2021).

In the next article “*Effect of disease severity on the structure and diversity of the phyllosphere microbial community in tobacco*” by Sun et al., amplicon sequencing-based analysis of ITS and 16S rRNA markers was employed to investigate the structure of microbiomes involved in tobacco target spot disease. The study revealed significant differences in the composition and diversity of phyllospheric fungi and bacteria between healthy plants and plants with varying levels of disease severity. In addition, critical fungal and bacterial biomarkers were identified for health and disease plants. High-throughput sequencing-based techniques such as amplicon sequencing and shotgun metagenomic sequencing are powerful tools that alleviate the limitations of traditional microbe separation techniques and can provide a complete picture of the microbial communities in samples of interest. These approaches not only help to elucidate the mechanisms of plant-pathogen and pathogen-pathogen interactions, but can also facilitate the monitoring of disease development and the evaluation of disease management strategies.

The complexity of the pathogen-host interaction is not only reflected in the change of the composition of the pathogen mixture at different stages on the same plant, but also in the different gene expression profiles of the resistant and susceptible plant varieties responding to the same pathogen. Gong et al. in the article “*Transcriptional profiling of resistant and susceptible cultivars of grapevine (Vitis L.) reveals hypersensitive responses to Plasmopara viticola*” revealed that the resistant grape variety “Kober 5BB” has significantly different gene expression profiles from the susceptible variety “Zitian Seedless”, and Caspase-like proteases mediating plant hypersensitive response (HR) cell death is the key strategy in the process of grape defense against downy mildew. Besides HR, reactive oxygen species (ROS) and plant hormones like salicylic acid (SA) and jasmonic acid (JA) have been reported to be involved in plant defense (Sherif et al., 2016; Rahman et al., 2019; Gupta et al., 2020; Hu et al., 2022). Additionally, Xu et al. in the article “*Mutations in PpAGO3 Lead to Enhanced Virulence of Phytophthora parasitica by Activation of 25–26 nt sRNA-Associated Effector Genes*” examined the *AGO3* gene of *Phytophthora parasitica*, and revealed an underappreciated role of small RNA (sRNA) silencing pathways in regulating the expression of effectors in pathogenic oomycetes.

In conclusion, this special issue focusing on pathogen co-Infections of plant diseases highlights the importance of the interactions between multiple pathogens and plant-pathogen and calls for future endeavors in this area. The underlying mechanisms resulting from pathogen co-infection that can lead to complex disease epidemiology are important topics for investigation and for the development of novel disease management approaches.

## Author contributions

JZ, SK, and XZ contributed to the manuscript preparation and finalizing. All authors contributed to the article and approved the submitted version.
